# Nkx2.5+ Cardiomyoblasts Contribute to Cardiomyogenesis in the Neonatal Heart

**DOI:** 10.1038/s41598-017-12869-4

**Published:** 2017-10-03

**Authors:** Vahid Serpooshan, Yuan-Hung Liu, Jan W. Buikema, Francisco X. Galdos, Orlando Chirikian, Sharon Paige, Sneha Venkatraman, Anusha Kumar, David R. Rawnsley, Xiaojing Huang, Daniël A. Pijnappels, Sean M. Wu

**Affiliations:** 1 Stanford Cardiovascular Institute, Stanford University School of Medicine, Stanford, CA USA; 2 Cardiovascular Research Center and Department of Medicine, Massachusetts General Hospital, 185 Cambridge Street, Boston, MA 02114 USA; 3 Division of Cardiology, Department of Medicine, Massachusetts General Hospital, 185 Cambridge Street, Boston, MA 02114 USA; 4 Section of Cardiology, Cardiovascular Center, Far Eastern Memorial Hospital, New Taipei City, Taiwan; 5 Department of Cardiology, University Medical Center Utrecht, Utrecht, The Netherlands; 6 Biology Program, California State University Channel Islands, Camarillo, CA USA; 7 Department of Pediatrics, Division of Pediatric Cardiology, Stanford University School of Medicine, Stanford, CA USA; 8 Department of Cardiology, Leiden University Medical Center, Leiden, The Netherlands; 9 Department of Medicine, Division of Cardiovascular Medicine, and Stanford University School of Medicine, Stanford, CA USA; 10 Institute of Stem Cell Biology and Regenerative Medicine, Stanford University School of Medicine, Stanford, CA USA

## Abstract

During normal lifespan, the mammalian heart undergoes limited renewal of cardiomyocytes. While the exact mechanism for this renewal remains unclear, two possibilities have been proposed: differentiated myocyte replication and progenitor/immature cell differentiation. This study aimed to characterize a population of cardiomyocyte precursors in the neonatal heart and to determine their requirement for cardiac development. By tracking the expression of an embryonic Nkx2.5 cardiac enhancer, we identified cardiomyoblasts capable of differentiation into striated cardiomyocytes *in vitro*. Genome-wide expression profile of neonatal Nkx2.5+ cardiomyoblasts showed the absence of sarcomeric gene and the presence of cardiac transcription factors. To determine the lineage contribution of the Nkx2.5+ cardiomyoblasts, we generated a doxycycline suppressible Cre transgenic mouse under the regulation of the Nkx2.5 enhancer and showed that neonatal Nkx2.5+ cardiomyoblasts mature into cardiomyocytes *in vivo*. Ablation of neonatal cardiomyoblasts resulted in ventricular hypertrophy and dilation, supporting a functional requirement of the Nkx2.5+ cardiomyoblasts. This study provides direct lineage tracing evidence that a cardiomyoblast population contributes to cardiogenesis in the neonatal heart. The cell population identified here may serve as a promising therapeutic for pediatric cardiac regeneration.

## Introduction

The mammalian heart possesses a limited capacity for new cardiomyocyte (CM) formation after embryonic development^1,2^. The basal cardiomyocte renewal rate in human hearts has been estimated to about 1% annually in the neonate and 0.45% in adults^1^. However, these estimates vary greatly among studies, some describing annual cardiomyote turnover as high as 20%^2^. While the exact extent of CM renewal continues to be debated, two possibilities have been proposed as possible mechanisms for their renewal: 1) new CMs are generated in the postnatal heart from the proliferation of pre-existing, differentiated CMs^3–8^; 2) a rare population of cardiac stem/progenitor cells contribute to normal cardiac development and tissue renewal during injury repair^9–12^.

The relative contribution by these distinct mechanisms to postnatal cardiomyogenesis has not been easily resolved due to the lack of an approach to identity the primitive cell population responsible for renewal. Until recently, there has been little data demonstrating that a postnatal cell population contributes directly to neocardiomyogenesis via cell labeling and lineage tracing in a prospective fashion^9,13,14^. While retrospective labeling studies confirm that cardiac stem/progenitor cells may contribute to endogenous cardiac renewal after injury^9^, this approach does not allow the identification of the precise cell population and the phenotypic characteristics they possess. Hence, this lack of a defined precursor cell population in the neonatal heart that can mediate *de novo* cardiomyogenesis has limited current efforts in cardiac regenerative therapy^15–18^.

To identify a population of CM precursors that might be present in neonatal heart, we utilize a previously generated transgenic mouse model that expresses an eGFP reporter under the regulatory control of a 2.1 kb cardiac-specific enhancer of Nkx2.5, a key transcription factor in early cardiac development^19^. Distinct from the endogenous expression of Nkx2.5, which is initiated in cardiac progenitor cells and sustained throughout CM maturation, the eGFP expression in Nkx2.5 cardiac enhancer-eGFP transgenic mice (hereto referred as Nkx2.5 enh-eGFP) is restricted to cardiac progenitor cells and early immature CMs^19,20^. Consequently, Nkx2.5 enh-eGFP+ cells represent cardiac progenitor cells in the early fetal heart and we postulate that it may also label a population of cardiomyogenic precursors in the postnatal heart.

Cardiac progenitor cells, such as the Islet-1 (Isl-1)-positive cell population, has been described in the neonatal heart^21^. However, the direct contribution of Isl-1+ cells to cardiomyogenesis in the postnatal heart *in vivo* has not been demonstrated^22,23^. Given the cardiomyoblast-restricted expression of Nkx2.5 enh-eGFP transgene in the fetal heart, we explored whether a rare number of these cells may be present in the neonatal heart and contribute to normal development of the myocardium. In this study we identified a neonatal Nkx2.5 enh-eGFP+ cardiomyoblast population and demonstrated their phenotypic and functional contribution to making new CMs. We further showed, by prospective lineage tracing using a doxycycline suppressible Nkx2.5 enhancer-Cre transgenic mouse line, that Nkx2.5 enh-eGFP+ cardiomyoblasts reside in the subepicardium and contribute directly to cardiomyogenesis *in vivo*. Furthermore, the ablation of neonatal Nkx2.5 enh-eGFP+ cardiomyoblasts led to early heart failure phenotypes, including ventricular dilation and hypertrophy, consistent with a requirement for these cells in normal neonatal heart development.

## Results

### Isolation and *in vitro* characterization of a putative cardiomyoblast population in the neonatal heart

**Figure 1:**
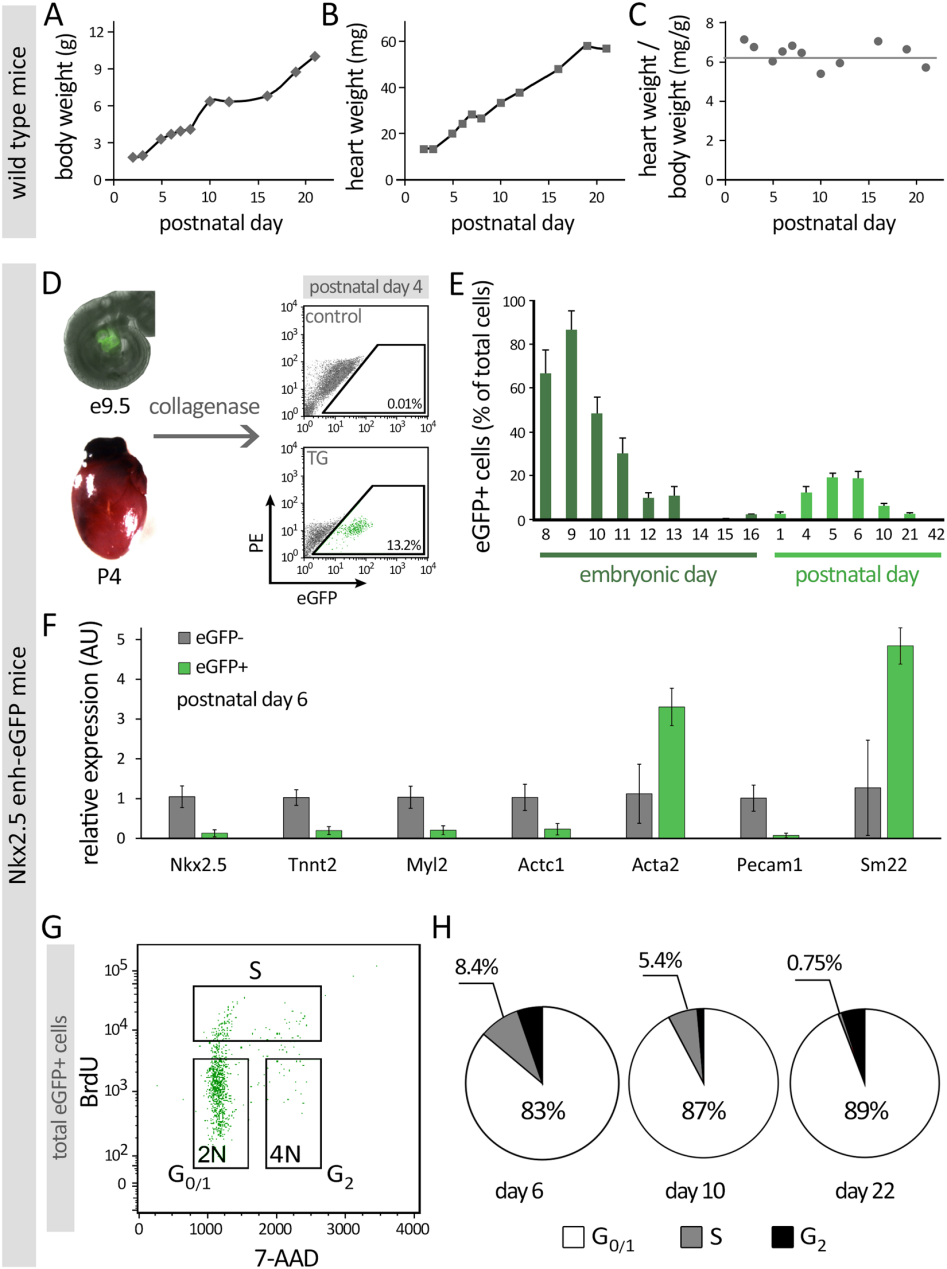
Increase in neonatal heart-body weight. Wild-type C57/BL6 neonatal mice (n = 3/time point) were sacrificed at the indicated time point and their body **(A)** and heart **(B)** weights were measured. The ratio of heart weight to body weight remained relatively constant during the first 3 weeks after birth **(C)**. **(D)** Schematic diagram of flow cytometric analysis of eGFP + cells from developing and neonatal (P4) Nkx2.5 enh-eGFP hearts. **(E)** Quantification of the percentage of eGFP + cells in embryonic hearts and in the non-myocyte fraction of neonatal hearts (n = 5/time point). **(F)** Quantitative PCR analysis of gene expression in FACS-purified GFP + (green) and GFP- (grey) cell populations (n = 5). Note that CM-associated cells localized predominantly in the eGFP- population. **(G-H)** *Identification of proliferating Nkx2.5 enh-eGFP + cells in the neonatal heart*. **(G)** Cell cycle analysis of eGFP + cells from day 6, 10, and 22 neonatal hearts (n = 3/time point). Shown is a representative flow cytometry plot of BrdU and 7-AAD incorporation into eGFP + cells. (**H**) The proportion of eGFP + cells in G_0/1_ (white), S (grey), and G_2_ (black) phases of cell cycle. The % of cells that are in G_0/1_ or S phases are indicated.

To determine the growth rate of the neonatal heart and its relationship with the growth of the overall body weight, we measured the heart weight and body weight in neonatal mice from birth to 21 days of life. We found a rapid rise in heart weight during this time period. The ratio of heart weight to body weight appeared to be stable during this developmental time frame (Fig. 1A–C). This finding demonstrated that a rapid growth occurs in the developing heart after birth. We hypothesized that postnatal cardiomyoblasts may contribute to the proliferating cells in the neonatal heart. Previously described Nkx2.5 enh-eGFP transgenic mice were used to isolate and characterize these cells^19,20^. The expression of eGFP in Nkx2.5 enh-eGFP mice labels cardiac precursor cells in the developing embryo and wanes when these cells mature into striated CMs^20^. Interestingly, by flow cytometric analysis of neonatal hearts from Nkx2.5 enh-eGFP mice, we found a resurgence of eGFP+ cell population during the first three weeks after birth (Fig. 1D,E).

qPCR analysis of sorted eGFP+ and eGFP- cells at postnatal day 6 demonstrated significant differences in the gene expression profiles of these cells (Fig. 1F). The proliferation capacity of the eGFP+ cells was quantified by determining the proportion of these cells in S or G_2_ phase of the cell cycle as measured by their incorporation of the DNA analog BrdU vs their DNA content (7-AAD) (Fig. 1G,H). We found that the proportion of eGFP+ cells in S phase declined from 8.4% at postnatal day 6 to 0.75% at day 22 while the proportion in G_0/1_ phase increased from 83% at day 6 to 89% at day 22. P6 eGFP+ were also isolated, cultured *in vitro*, and immunostained for proliferation markers including Ki67 and pH3 at days 1 and 5 in culture (Supplementary Figure S1). Confocal microscopy demonstrated a notable percentage of eGFP+ cells were Ki67+ (~30% at day 1 and 20% at day 5) and pH3+ (~18% at day 1 and 12% at day 5).

**Figure 2:**
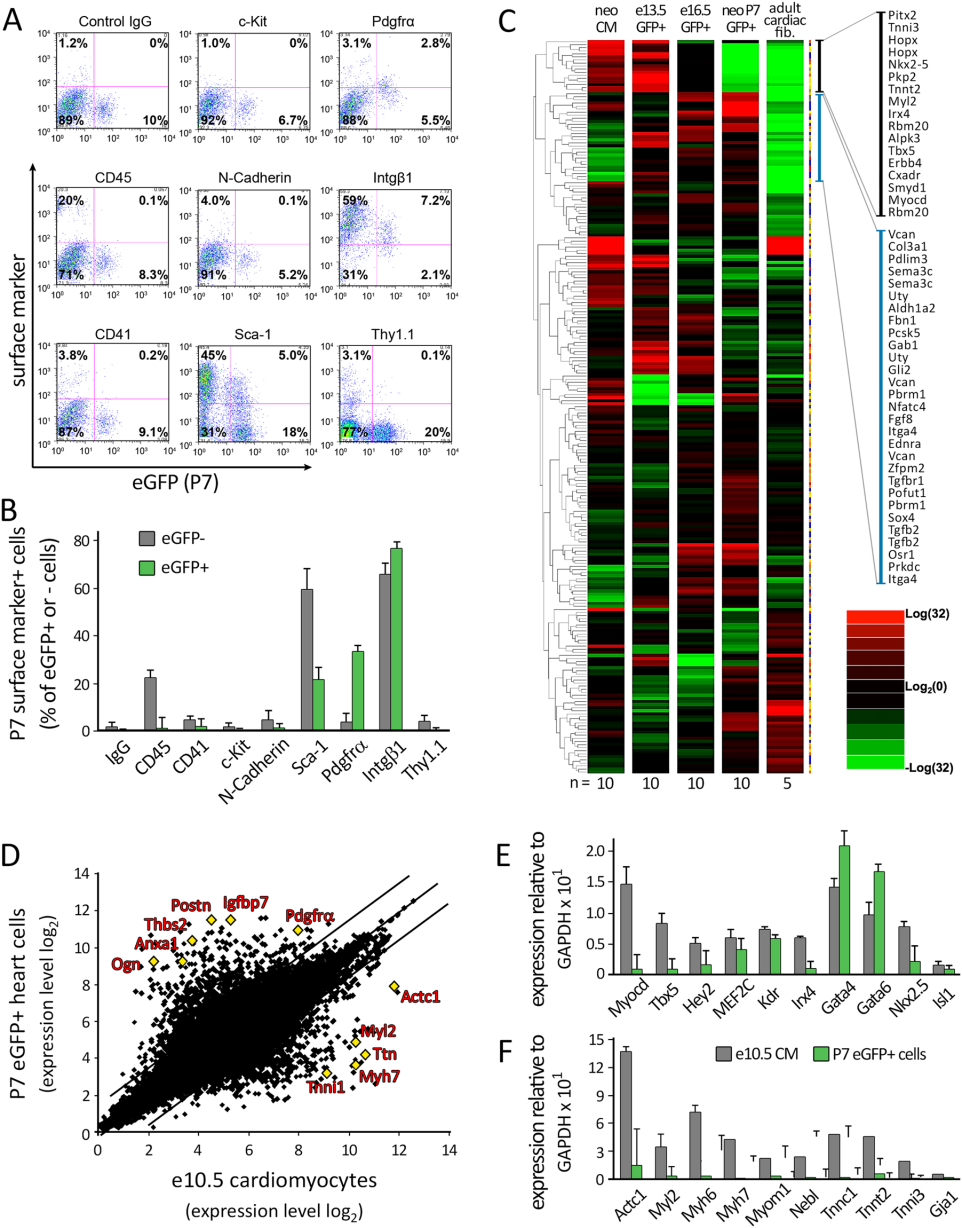
Phenotypic characterization of neonatal Nkx2.5 enh-eGFP + cells. **(A)** Analysis of surface marker expression in eGFP + and eGFP- non-myocytes from the neonatal Nkx2.5 enh-eGFP heart. Shown are representative flow plots (n = 4). **(B)** Quantification of the percentage of eGFP + and eGFP- cells that express the indicated surface marker. **(C)** Genome-wide expression analysis of embryonic day 13.5 (n = 10), 16.5 (n = 10), and neonatal P7 (n = 10) (total) GFP + cells against neonatal CMs (neo CM) (n = 10) and adult cardiac fibroblasts (n = 5). **(D)** Comparison of genome-wide transcriptional profile of neonatal eGFP + cells with embryonic day 10.5 (e10.5) CMs, isolated from wild type mice. **(E,F)** Quantitative PCR analysis of cardiac transcription factor **(E)** and sarcomeric gene **(F)** expression in neonatal day 7 (P7) eGFP + cells and e10.5 CMs.

We further characterized these (total) neonatal eGFP+ cells by flow cytometric analysis to determine their surface marker expression. About 33.7% of these cells specifically expressed PDGF receptor alpha (Pdgfr*α*), ~76.8% non-specifically expressed integrin beta-1 (Intg*β*1), and ~22.0% expressed stem cell antigen-1 (Sca-1) (Fig. 2A,B). Moreover, these cells did not express CD45, a pan-hematopoietic marker; Thy1.1, a mesenchyme/ fibroblast marker; or hematopoietic stem/progenitor cell markers such as CD41 or c-Kit. Given that Pdgfr*α* has previously been described as a fibroblast or mesenchymal stem cell marker in the adult heart^24,25^, we compared the genome-wide transcriptional profile of eGFP+ cells isolated at embryonic days 13.5 (e13.5 GFP+ ) and 16.5 (e16.5 GFP+ ) of development and from neonatal heart (neo P7 GFP+ ) with control neonatal CMs (neo CM) and cardiac fibroblasts from the adult heart (adult cardiac fib.) (Fig. 2C). Neonatal P7 eGFP+ cells expressed a distinct transcription profile from embryonic eGFP+ cells, neonatal CMs, or cardiac fibroblasts. To further probe the identity of these neonatal eGFP+ cells, we compared directly the genome-wide expression profile of embryonic day 10.5 (e10.5) CMs with P7 eGFP+ cells (Fig. 2D). The expression profile of P7 eGFP+ cells appeared quite distinct from that of e10.5 CMs. This was further supported by quantitative RT-PCR analysis showing that P7 eGFP+ cells express a number of cardiac transcription factors (*e.g*. MEF2C, GATA4, and GATA6) without a matching level of sarcomeric gene expression (*e.g*. troponin C1, T2, I3, cardiac actin, myl2, and myh6 and 7) (Fig. 2E,F).

### Neonatal Nkx2.5 enh-eGFP+ cells possess the functional characteristics of neonatal cardiomyoblasts

**Figure 3:**
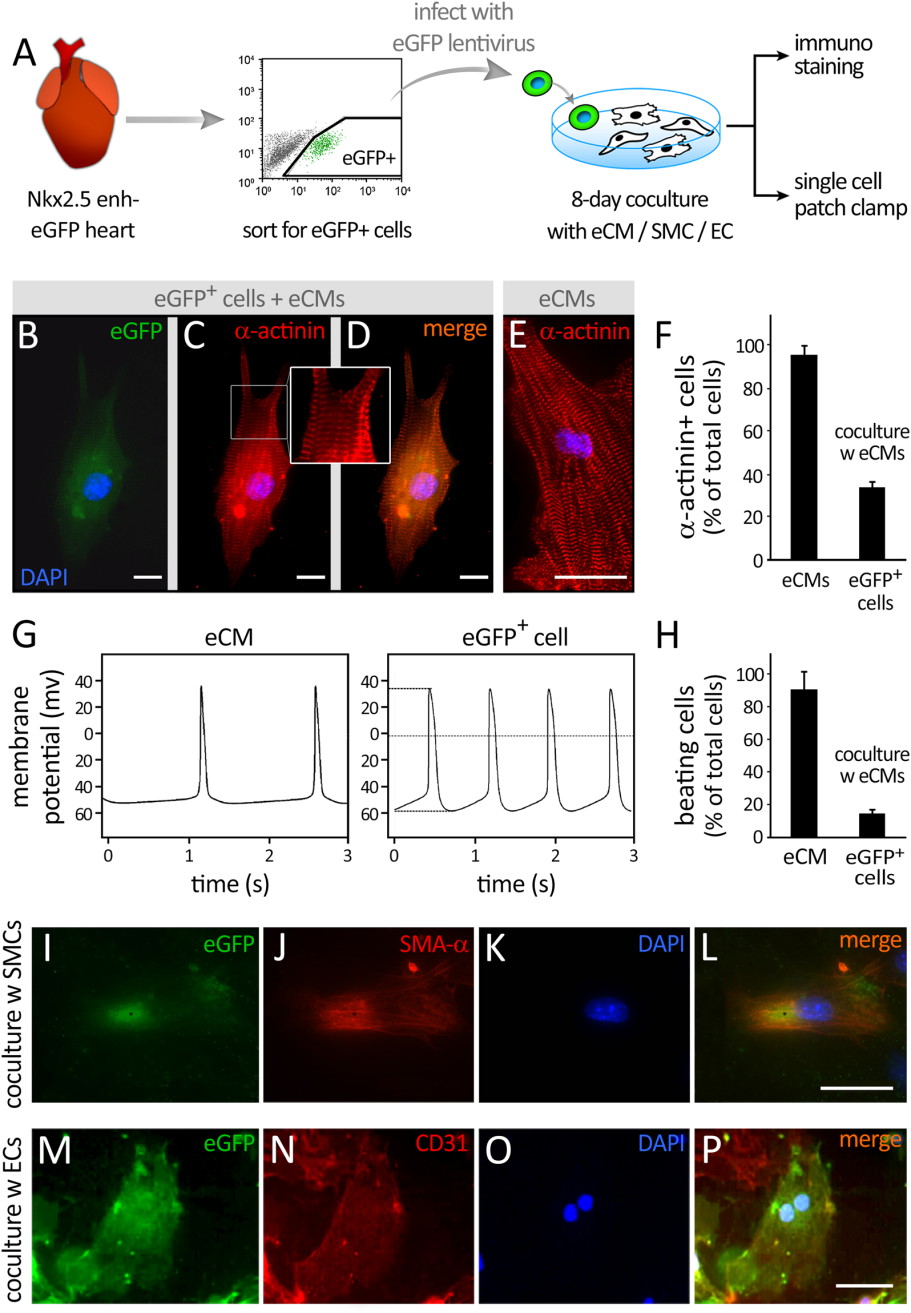
Functional characterization of neonatal Nkx2.5 enh-eGFP + cells. **(A)** Schematic illustration of *in vitro* differentiation of FACS-purified neonatal eGFP + cells into cardiomyocyte (CM), smooth muscle cell (SMC), and endothelial cell (EC), in coculture with embryonic day 10.5 CMs (eCMs), aortic SMCs, and endothelial cells, respectively (n = 5). **(B**–**E)** Immunofluorescent costaining for eGFP and α-sarcomeric actinin at 8 days after coculture followed by collagenase treatment and single cell re-plating. Scale = 20 µm. The inset in panel C shows a magnified view of sarcomeric structure in differentiated CM. **(F)** Quantification of the percentage of eCMs and cocultured eGFP + cells expressing α-sarcomeric actinin (n = 5). **(G,H)** Electrophysiological assessment of a control eCM and single re-plated eGFP + cell. The percentage of cells capable of spontaneous beating is shown (n = 33). **(I**–**L)** Immunofluorescent co-staining for eGFP and smooth muscle actin-α (SMA-α) at 8 days after coculture (n = 5). **(M**–**P)** Immunofluorescent co-staining for eGFP and CD31/PECAM at 8 days after coculture (n = 5). Scale bars = 20 µm.

To address whether these neonatal Nkx2.5 enh-eGFP+ cells harbor a capacity for cardiovascular lineage differentiation, eGFP+ cells were isolated from 6-7 day-old hearts, FACS-purified, and subjected to either spontaneous differentiation or differentiation in coculture with embryonic CMs (eCMs), smooth muscle cells (SMCs), mouse embryonic fibroblasts (MEFs), or endothelial cells (ECs) (Fig. 3 and Supplementary Figure S2). eGFP+ cells cultured alone expressed little to no cardiac troponin T, whereas eGFP+ cells cocultured with eCMs for 8 days expressed both cardiac troponin T (~51% of cells, Supplementary Figure S2B,C, top row) and sarcomeric actinin (~28% of cells) and adopted a striated CM phenotype (Fig. 3B–F). Single cell electrophysiological assessment of an eCM-cocultured and subsequently disbursed eGFP+ cell revealed its ability to generate spontaneous action potentials (Fig. 3G,H). Interestingly, when neonatal eGFP+ cells were cocultured with aortic SMCs instead, a significant proportion of the cells expressed smooth muscle myosin heavy chain (SM-MHC) (Supplementary Figure SB-C, middle row) and smooth muscle actin-*α* (SMA-*α*) (Fig. 3I–L), and adopted smooth muscle cell morphology. This indicates that paracrine factors and/or cell-cell contact may play an important role in the conversion of eGFP cardiomyoblasts into functional CMs. On the other hand, coculturing of neonatal eGFP+ cells with endothelial cells minimally increased their expression of CD31 (Fig. 3M–P and Supplementary Figure S2B,C, bottom row), in support of their pre-existing commitment to myogenic lineages.

### Differentiation of Nkx2.5 enh-eGFP+ cells into CMs *in vivo*

**Figure 4:**
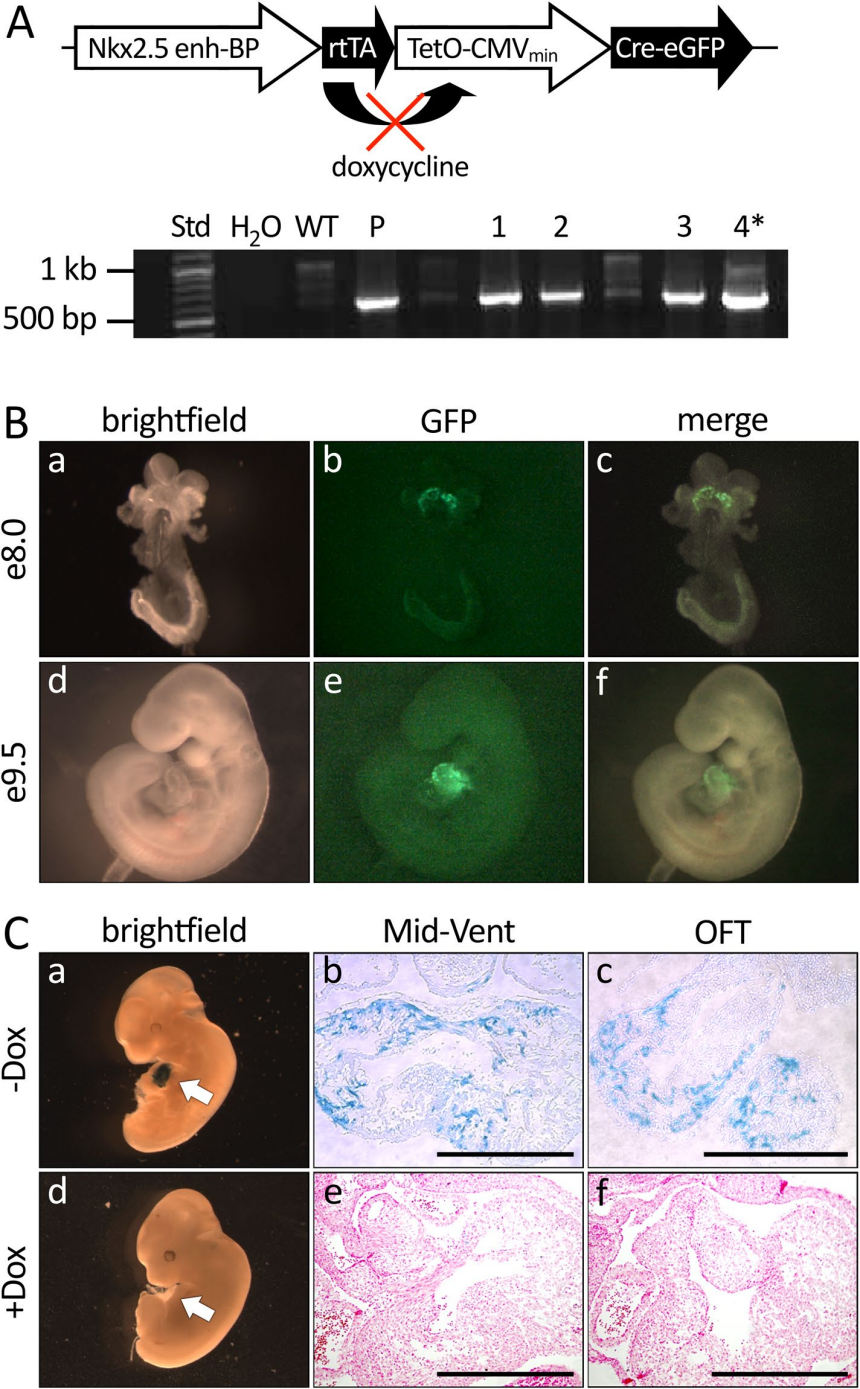
Generation and characterization of doxycycline-regulated Nkx2.5 enhancer-Cre transgenic mice. **(A)** Diagram of the plasmid construct used to generate the transgenic mice (top panel). Bottom panel: confirmation of the presence of transgenic sequence in founder mice by genomic PCR analysis. **(B)** Whole-mount fluorescence microscopy of Nkx2.5 enh-Cre embryos at days 8.0 and 9.5 post coitum (n = 2). Note the cardiac-restricted expression of Cre-eGFP. **(C)** Analysis of Cre + cells and their lineage descendants in Nkx2.5 enh-Cre /ROSA26^FS^LacZ transgenic embryos (n = 3) at 12.5 day post coitum in the absence (a,b,c) or presence (d,e,f) of doxycycline administration. (a,d) Whole mount bright field microscopy of X-Gal stained embryos. (b,c,e,f) Transverse sections of X-Gal stained embryos at day 12.5 post-coitum. Mid-Vent: Mid-ventricular. OFT: outflow tract.

To address whether neonatal eGFP+ cells are able to expand and differentiate into mature CMs *in vivo*, we engineered a transgenic mouse line that expresses a Cre-eGFP fusion protein under the control of both Nkx2.5 cardiac enhancer and the reverse tetracycline transactivator (hereto referred as *Nkx2.5 enh-Cre mouse*) (Fig. 4A). By oral administration of doxycycline, the Cre-eGFP fusion protein expression can be silenced, thus providing a temporal level of gene regulation. Similar to the Nkx2.5 enh-eGFP transgenic embryos at the same stages of development, the expression of eGFP in the Nkx2.5 enh-Cre transgenic embryos was restricted to the developing heart (Fig. 4B). When the Nkx2.5 enh-Cre mice were mated with the ROSA26^FS^LacZ reporter mice, the double transgenic embryos exhibited cardiac-specific LacZ labeling (Fig. 4Ca–c). With gestational administration of doxycycline, the embryonic LacZ expression was completely abolished (Fig. 4Cd–f).

**Figure 5:**
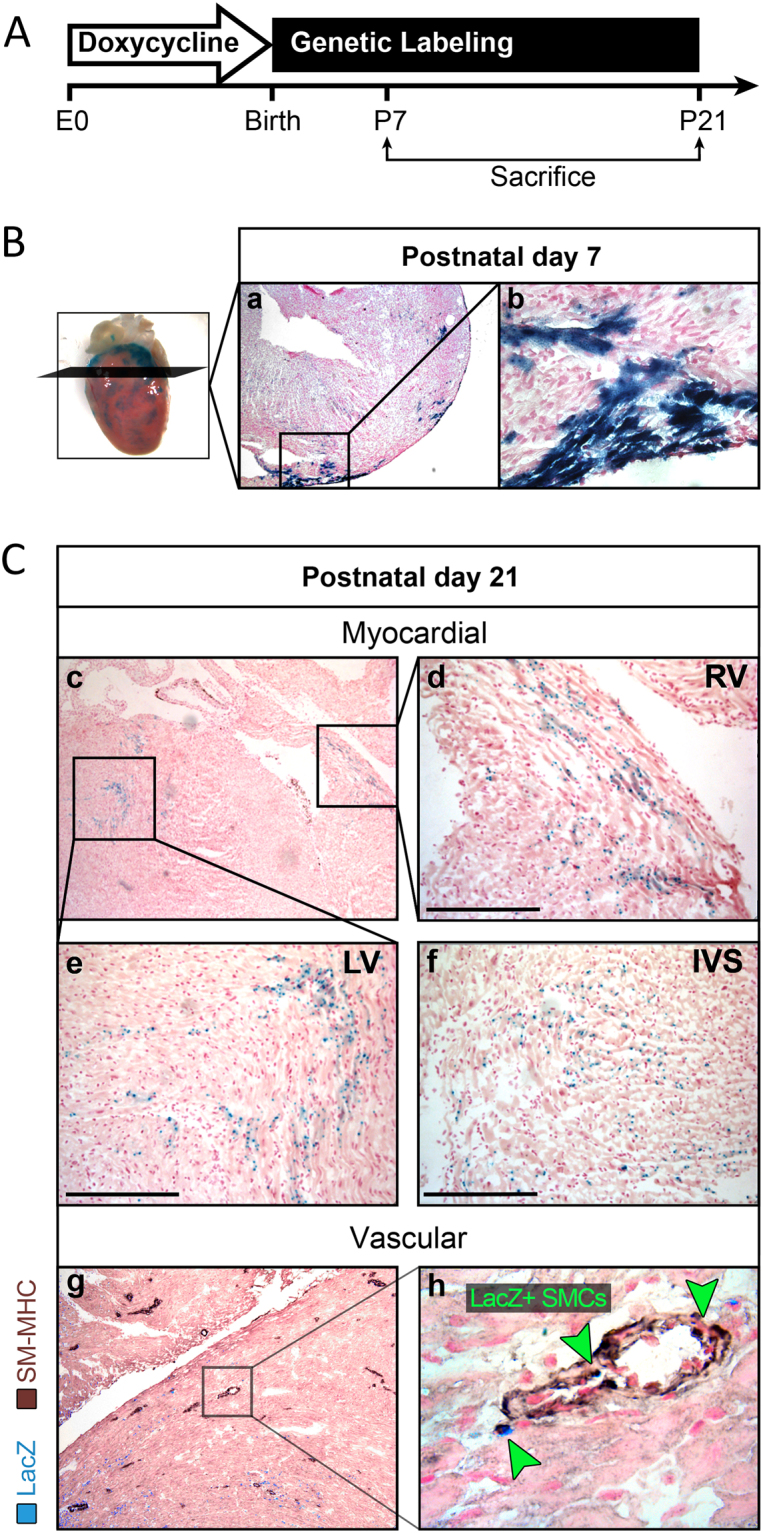
Lineage labeling of neonatal cardiomyoblasts using doxycycline-regulated Nkx2.5 enhancer-Cre transgenic mice **(A)** Diagram of the experimental strategy to lineage label neonatal cardiomyoblasts and their progenies. **(B,C)** Detection of lineage descendants of neonatal Cre + cells in the hearts of Nkx2.5 enh-Cre/ROSA26^FS^LacZ mice at day 7 (n = 3) **(B)** and 21 (n = 3) **(C)** after birth by X-gal staining.

The ability to completely silence Nkx2.5 enh-Cre expression during embryonic development allowed us to determine whether the neonatal eGFP+ cardiomyoblasts were able to contribute to CM formation in the neonatal heart. To address this, we treated pregnant Nkx2.5 enh-Cre females that were mated with ROSA26^FS^LacZ males with doxycycline from conception until birth to suppress the embryonic expression of Cre-eGFP (Fig. 5A) and fully label Nkx2.5 enh-eGFP+ cells from P4 onwards when the doxycycline suppression of Cre expression is completely lost. We then assayed for the presence of LacZ+ cells at postnatal day 7 and 21 to determine whether neonatal Nkx2.5 enh-eGFP+ cardiomyoblasts had given rise to new CMs in the neonatal heart. We found that the Cre+ cardiomyoblasts and their descendant CMs were located in the subepicardial region in the neonatal heart at day 7 after birth (Fig. 5B). By day 21, many of these cells had migrated and differentiated into mature CMs and could be identified in the right and left ventricles (RV, LV) as well as in the interventricular septum IVS (Fig. 5C, c–h). Interestingly, sparse LacZ+ coronary SMCs were also be found within the vessel walls (Fig. 5C, g,h).

### Embryonic origin of Nkx2.5 enh-eGFP+ cardiomyoblast

The subepicardial localization of new CMs from neonatal Nkx2.5 enh-eGFP+ cardiomyoblasts raises the possibility that the Nkx2.5 enh-eGFP+ cardiomyoblasts might have originated from the developing epicardium. This would be consistent with recent studies showing the ability of developing and postnatal epicardial cells to differentiate into CMs^26–28^. We investigated whether subepicardial cardiomyoblasts in the neonatal heart originated from embryonic epicardial cells using a previously described inducible WT1-CreERT2 mice^28^ and found no evidence that these cells came from the developing epicardium (Supplementary Figure S3). This is consistent with results from a recent lineage tracing study of postnatal cardiac regeneration in the zebrafish heart^6^. We further examined whether neonatal cardiomyoblasts descended from other precursor populations such as endothelial/endocardial (Tie2-Cre) or mature myocardial (alpha-myosin heavy chain-Cre) (αMHC-Cre) cell populations. No lineage relationship was found between developing endothelial/endocardial cells or mature CMs and the neonatal eGFP+ cardiomyoblasts. Instead, we show that neonatal eGFP+ cells are descendants from embryonic Nkx2.5 enh-eGFP+ cells in the fetal heart (Supplementary Figure S3).

### Nkx2.5 enh-eGFP+ cardiomyoblast-mediated cardiomyogenesis in the neonatal heart is developmentally significant

**Figure 6:**
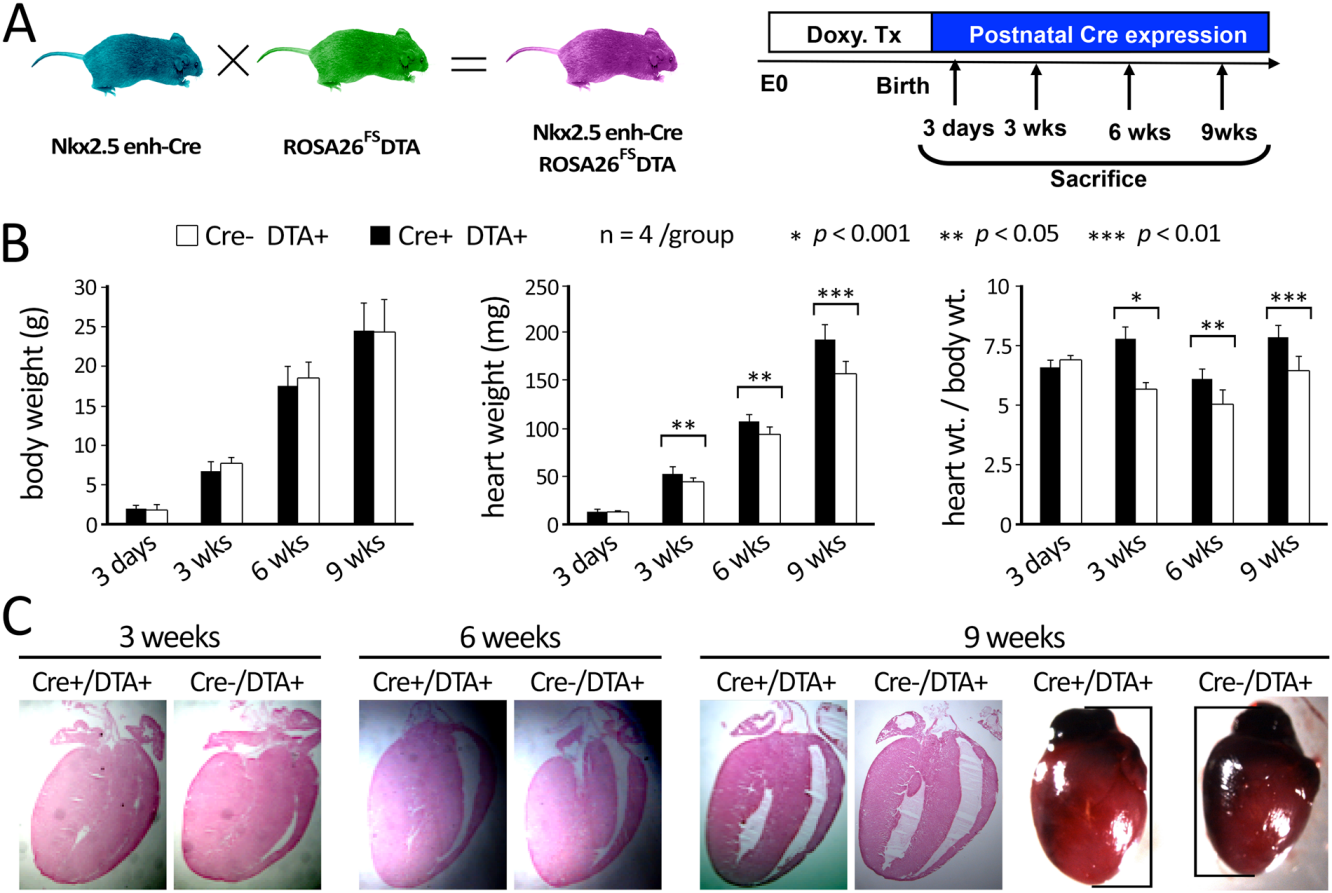
Phenotype of neonatal mice following the deletion of Nkx2.5 + cardiomyoblasts. **(A)** Breeding and doxycycline treatment strategy for Cre/LoxP-mediated ablation of neonatal Nkx2.5 + cardiomyoblasts. The excision of the stopper cassette results in diphtheria toxin A (DTA) expression in Cre-expressing cells. **(B)** The hypertrophic response of neonatal mouse hearts that have undergone ablation of their Nkx2.5 + cardiomyoblasts shortly after birth. The body weight (left panel), heart weight (middle panel), and the ratio of heart weight to body weight (right panel) are shown (n = 4 for each time point). **(C)** Histological examination of hearts in mice with deficiency of Nkx2.5 + cardiomyoblasts. At 9 weeks old, the cardiomyoblast-deficient hearts (Cre + /DTA + ) are larger in size than that of their littermate control (Cre-/DTA+ ) (right panel).

To determine the consequences of the loss-of-function of Nkx2.5 enh-eGFP+ cardiomyoblasts during neonatal heart formation, we treated compound heterozygous Nkx2.5 enh-Cre;ROSA26^FS^DTA mouse embryos with doxycycline from conception until birth to suppress embryonic Cre expression (Fig. 6A). The ROSA26^FS^DTA mouse expresses diphtheria toxin upon Cre-mediated excision of the LoxP flanked stopper cassette^29^. The expression of Cre upon the cessation of doxycycline administration at birth results in excision of the stopper cassette in Cre+ cells, the production of DTA, and shortly after, the death of Nkx2.5 enh-Cre+ cells. As shown in Fig. 6B, mice with ablation of neonatal cardiomyoblasts (Cre+ /DTA+ mice – black bar) exhibited increased heart weight at 3, 6, and 9 weeks after birth compared with their littermate control (Cre-/DTA+ mice – white bar), without significant difference in their body weight (Fig. 6B). This suggests that ablation of neonatal Nkx2.5+ cardiomyoblasts leads to early remodeling changes including ventricular hypertrophy. With further maturation (at 9 weeks), the ablated hearts exhibit mild ventricular enlargement as well (Fig. 6C). Ablation of Nkx2.5 enh-eGFP+ cell population did not compromise mice viability or health.

## Discussion

The neonatal heart grows rapidly in both size and weight in order to meet the metabolic demands of the newborn. Beyond the first one or two weeks after birth, these increases in heart size and weight are thought to be mediated entirely by myocyte hypertrophy rather than proliferation^30–32^. In this study, we found a population of Nkx2.5 enhancer+ cardiomyoblasts in the neonatal heart that can differentiate into striated CMs upon coculture with embryonic CMs. These cells are found initially in the subepicardial region and contribute progressively to new CMs in the right and left ventricles as well as the interventricular septum. Genetic ablation of these cells using a conditional diphtheria toxin A-expressing mice results in early heart failure phenotype. These data support the contribution of cardiomyoblasts to a defined proportion of the proliferating CMs in the neonatal heart and the requirement for CM proliferation to support normal neonatal heart development^33,34^.

The reappearance of eGFP+ cells in the neonatal heart of Nkx2.5 enh-eGFP mice suggested a potential contribution of cardiomyoblasts to the postnatal proliferative activity (Fig. 1G). qPCR analysis of isolated GFP+ and GFP- cells from the P6 neonatal mouse heart (Fig. 1F) demonstrated significantly lower Nkx2.5 expression level in the GFP+ cells, suggesting that the presence of the fetal enhancer activity in GFP+ cells does not precisely correlate with higher endogenous Nkx2.5 gene expression in postnatal cells. This can be explained by the fact that multiple enhancers are involved in controlling the activity of endogenous Nkx2.5 expression and other Nkx2.5 enhancers are more dominant in cardiac cells (e.g. cardiomyocytes) in the postnatal heart^35^. This can be due to the fact that different enhancers control the activity of Nkx2.5 expression and that in Nkx2.5 transcription itself is not specific to progenitors. The low expression levels of cardiac and endothelial genes in eGFP+ cells are line with our finding that these eGFP+ cells are mostly undifferentiated multipotent progenitors.

BrdU pulse-labeling of isolated Nkx2.5 enh-eGFP+ cells confirmed their residual proliferative activity that continued for a period (until P21) longer than previously described^36,37^. This data indicated that the proliferative capacity of eGFP+ cells was the greatest shortly after birth and declined over the first 3 weeks of life (Fig. 1J). The *in vitro* culture of P6 isolated eGFP+ cells confirmed their proliferative capacity (Ki67 and pH3 immunostaining) which declined from day 1 to day 5, while the GFP- cells maintained their expression levels (Supplementary Figure S1). Phenotypic characterization of neonatal Nkx2.5 enh-eGFP+ cells, via both surface marker and genome-wide expression analysis, demonstrated remarkably distinct profile of these eGFP+ cells.

Our detailed characterization of the cellular phenotype of neonatal Nkx2.5 enhancer+ cardiomyoblasts revealed distinct properties of these cells from those described previously. Their expression of Pdgfr*α* (Fig. 2A,B) is consistent with their embryonic heart field origin given the previously reported labeling of embryonic^38^, postnatal^25^, as well as embryonic stem cell-derived^39^ cardiac precursors with this marker. However, our genome-wide expression analysis revealed their distinct characteristic from either cardiac fibroblasts or embryonic CMs (Fig. 2C–F). It is worth noting that these eGFP+ cells exhibit a high level of expression of signaling molecules (*e.g*. Fgf8, Tgf-β2, Tgf-βR1, Gab1, Sema3C, Ednra) and transcription factors (e.g. Zfpm2, Nfatc4, Gli2, Pbrm1/BAF180, Osr1) but not sarcomeric genes in comparison to that of fetal CMs (Fig. 2B,F). All together, the cell marker and genome-wide expression profiles of these neonatal Nkx2.5 enhancer+ cells are consistent with a cell population that is distinct from cardiac fibroblasts or mature CMs and suggests their role as a cardiomyoblast population.

Through a series of coculture experiments, we demonstrated the ability of neonatal Nkx2.5 enh-eGFP+ cells, cocultured with eCMs, to differentiate into striated CMs, expressing CM-specific troponin T and sarcomeric actinin (Fig. 3 and Supplementary Figure S2). In the ROSA-LacZ heart, it appeared that the expression of *β*-gal in each cell may be variable as a few ( <10%) of cells were weakly *β*-gal positive (Supplementary Figure S2). This could be due to the fact that the *β*-gal expression from the ROSA26 locus is not uniformly strong in all cells in postnatal tissue. This differentiation of eGFP+ cells into CMs depends on paracrine and/or contact factors since little to no cardiac troponin T+ cells was generated from spontaneous differentiation of eGFP+ cells. The cardiomyogenic phenotype of eCM-cocultured eGFP+ cells was not due to cell-cell fusion since the majority of sarcomeric actinin/eGFP double positive cells exhibited only one single nucleus (Fig. 2B–D). Consistent with our previous reports^20^, the Nkx2.5 enh-eGFP+ cells demonstrated a remarkable capacity to differentiate into SMCs both *in vitro* (Fig. 3 and Supplementary Figure S2) and *in vivo* (Fig. 5). These findings provide strong support that neonatal Nkx2.5 enh-eGFP+ cells represent a population of cardiomyoblasts in the neonatal heart. For future studies, it would be of high interest to unravel the molecular pathways driving these cell-fate decisions to either become myocardium, smooth muscle or endothelium.

The capacity of Nkx2.5 + cardiomyoblasts in the neonatal heart to expand, differentiate, and mature into CMs *in vivo* raises an interesting question regarding their importance during neonatal cardiac development. Using the newly engineered Nkx2.5 enh-Cre mouse model, we found that Nkx2.5 + cardiomyoblasts gave rise to new CMs in the subepicardium that progressively migrated inward to contribute to new CMs in the right and left ventricles and interventricular septum (Fig. 5). This is while a small fraction of LacZ + cardiomyoblasts differentiated to SMCs, residing within the vessel walls (CM to SMC percentage ratio of ~87:13) (Fig. 5C,g,h). FACS analysis of the P0 isolated eGFP+ cells which were co-stained with cardiac Troponin T (cTnT) demonstrated a negligible fraction eGFP + cells expressing cTnT (~0.01% of total cells) (Supplementary Figure S4).

In support of the importance of these cells to normal cardiac development, we found that the ablation of these cells led to enlarged heart size, elevated heart/body weight ratio, and left ventricular hypertrophy that eventually dilate over time (Fig. 6C). This pattern is consistent with the progression of many pediatric cardiomyopathies and suggests that modulation of cardiomyoblast proliferation and differentiation may be therapeutically relevant in this patient population^40^. Further analyses (*e.g*., echocardiography or immunohistochemistry of cardiac disease markers) would be required to achieve a greater understanding of the role of Nkx2.5 enh-eGFP+ cardiomyoblasts in maintaining the function of developing heart. Moreover, we are currently investigating the potential role of Nkx2.5-enh-eGFP+ cells on the heart regenerative response in a myocardial infarction model in postnatal regenerative window (P0-P7), juvenile (P21), and adult (7–12 weeks old) mice. Taken together, these results support the requirement of neonatal Nkx2.5 enh-eGFP+ cardiomyoblasts to generate functional CMs during normal cardiac development.

## Methods

### Mice

Newborn wild type C57/BL6 mice (Jackson Laboratory, Barharbor, ME) were sacrificed at days 1–21 and their body and heart weights were measured. Euthanasia was performed by first sedating the mice via isoflurane (inhalant, 2% in 100% oxygen, neonate placed on a warm pad), followed by a secondary cervical dislocation^41^. Death was verified after euthanasia and prior to disposal. Nkx2.5 cardiac enhancer-eGFP transgenic mice (Nkx2.5 enh-eGFP) were previously described^20^. Doxycycline-regulated Nkx2.5 enhancer-Cre-eGFP transgenic mice (Nkx2.5 enh-Cre) were made by pronuclear injection of one-cell C57BL/5 mouse embryos and transferred to CD1 pseudopregnant foster females. From four original transgene-carrying founders, the line with the most robust expression of Cre-eGFP in the developing heart was further studied. ROSA26-flox-stop-flox-LacZ reporter mice (ROSA26^FS^LacZ) were obtained commercially from Jackson Laboratory (Bar harbor, ME). CM-specific alpha-myosin heavy chain-Cre (α-MHC-Cre), endothelial Tie2-Cre, and ROSA26-flox-eGFP-flox-diptheria toxin A (ROSA26^FS^DTA) mice were described previously^29,42,43^. All animal experiments were approved by the Subcommittee on Research Animal Care at Massachusetts General Hospital and by the animal care and use committee (APLAC) at Stanford University. All experiments were performed in accordance with relevant guidelines and regulations of Massachusetts General Hospital.

### Body and heart weight measurements

To determine the heart weight and body weight of neonatal mice, we sacrificed mice at the indicated age (by day) of development. Their overall body weight was measured and the hearts were dissected and cleared with deionized water and measured from day 1 to 21 after birth. In experiments involving transgenic mice that have undergone Cre-mediated ablation of neonatal Nkx2.5 + cardiomyoblasts, the body and heart weights were measured at 0.5, 3, 6, and 9 weeks after birth and compared with those of the littermate control without Cre transgene.

### Histology, immunohistochemistry, and immunofluorescence

Freshly isolated adult and embryonic mouse hearts were dissected from mouse chest cavity and washed in PBS to remove excess blood. For Nkx2.5 enh-eGFP and Nkx2.5 enh-Cre embryos at days 8.0 and 9.5 post coitum, their heart tube was dissected away from the body and imaged immediately with whole mount fluorescence microscopy. For late fetal and postnatal hearts, they were incubated in 30% sucrose in PBS overnight followed by step-wise incubation with a graded concentration of OCT in PBS for cryosectioning. Following cryopreservation, hearts were cut into 10 μm sections and lightly fixed in 4% paraformaldehyde in PBS prior to immunostaining. For detection of CM differentiation, antibodies against α-sarcomeric-actinin (1:200; Sigma-Aldrich, St. Louis, MO) and cardiac troponin-T (1:200; polyclonal, Chemicon) were used. For visualization, fluorescence detection with Alexa Fluor® secondary antibodies (Invitrogen, Carlsbad, CA) towards the appropriate primary antibodies was used. For β-galactosidase staining, freshly dissected mouse hearts were prepared as described above and incubated at 37 °C in 1 mg/ml X-Gal substrate (Fisher Scientific). The X-Gal stained sections were then counterstained with Nuclear Fast Red and/or co-stained with antibodies for co-immunofluorescent studies. Hematoxylin and eosin staining of histological sections was performed according to manufacturer suggested protocol. All quantitative analyses of the histological sections were performed on numerically-coded animals in an observer-blinded fashion to prevent subjective bias in data analysis.

### Cell isolation, flow cytometry, FACS sorting, and cell cycle analysis

At indicated age, freshly isolated hearts from embryonic and neonatal Nkx2.5 enh-eGFP transgenic mice were immediately minced and digested with collagenase (collagenase A 10 mg/ml and B 10 mg/ml, Roche, Sigma-Aldrich) in 10 mM HEPES buffered solution with 20% FBS at 37 °C for 60 min. Single-cell suspension was obtained by trituration every 15 min during incubation followed by removal of undigested tissue with a 40 μm cell strainer. eGFP+ live cells, as defined by propidium iodine negativity, were analyzed by flow cytometry using FACSCalibur® (BD Biosciences) or isolated using FACSAria® (BD Biosciences) and cultured in differentiation medium (DM) containing IMDM (Invitrogen), 20% FCS (Invitrogen), 5000 i.u./ml penicillin/streptomycin (Invitrogen), 200 mM L-glutamine (Invitrogen), 1.5 × 10^−4^M 1-thioglycerol (Sigma-Aldrich), and 50 μg/ml ascorbic acid (Sigma-Aldrich). For co-culture experiments, Nkx2.5 enh-eGFP+ sorted cells were first transfected with a GFP expressing lentivirus to permanently label these cells before their differentiation begins.

For cell cycle analysis, BrdU was injected into the peritoneum of neonatal (only once, at day 5 or 20) Nkx2.5 enh-eGFP+ and wild-type mice. At 5 hrs after injection (wild type) or 24 hrs after injection (Nkx2.5 enh-eGFP+ ) the hearts were harvested for collagenase treatment and flow cytometry analysis using a combination of 7-AAD and anti-BrdU-APC antibody (BD Biosciences). Flow cytometry and cell cycle data were acquired on a FACSCalibur® (BD Biosciences) flow cytometer and processed by FlowJo software (Treestar). The flow cytometry antibodies used were APC-conjugated anti-mouse CD4, CD8, CD45, Gr-1, Mac-1, B220, and c-Kit (from eBioscience) as well as PDGF receptor alpha (Pdgfr*α*), integrin beta-1 (Intg*β*1), stem cell antigen-1 (Sca-1), CD45, Thy1.1, and CD41 (from BD Biosciences).

### Preparation of neonatal CMs and adult cardiac fibroblasts

Hearts were extracted from neonatal mice (P7) and immediately transferred into the dish containing 1× PBS on ice and washed twice. Subsequently, heart were transferred into the isolation medium (20 mM BDM, 0.0125% trypsin, in HBSS) and minced into small pieces (on ice). Minced hearts were transferred into a tube containing isolation medium and incubated with gentle agitation at 4 °C overnight. Predigested hearts containing tissue fragments were further digested using collagenase (15 mg in 10 mL of L15 medium) at 37 °C for 20 minutes. Neonatal CMs were then strained (40 μm), centrifuged (300 rpm- 5 min), resuspended, and plated onto collagen-coated cell culture plates (Sigma C-8919) using plating medium (65% DMEM, 19% M-199, 15% fetal calf serum, 1% penicillin/streptomycin)^44^. To prepare cardiac fibroblasts, adult mouse hearts were extracted, washed twice with ice cold PBS, and minced on ice (~1 mm). Minced tissue was digested using collagenase (1% v/v collagenase II in HBSS buffer) under constant stirring at 37 °C for 20–30 min. Once fully digested, the supernatant was transferred to a tube (on ice) containing 1 ml fibroblast medium (DMEM/F12 with 10% FBS, 100 U/ml Pen/Strep, 1× L-glutamine, and 100 µM ascorbic acid), centrifuged (300 g, 5 min), and resuspend in fibroblast medium. Cardiac fibroblasts were plated into 10-cm cell culture dishes and incubated at 37 °C in a cell culture incubator with 5% CO_2_ for 2 hrs. Once fibroblasts adhered onto the dish, we discarded the supernatant, rinsed the cells with PBS (3x) and added fresh fibroblast medium^45^.

### Microarray analysis

The oligonucleotide microarrays were performed by WELGENE Microarray Service (Taiwan). 0.2 μg of total RNA was amplified by a Low Input Quick-Amp Labeling kit (Agilent Technologies, USA) and labeled with Cy3 (CyDye, Agilent Technologies, USA) during the *in vitro* transcription process. 0.6 μg of Cy3-labled cRNA was fragmented to an average size of about 50–100 nucleotides by incubation with fragmentation buffer at 60 °C for 30 minutes. Correspondingly fragmented labeled cRNA was then pooled and hybridized to Agilent SurePrint G3 Mouse GE 8 × 60 K Microarray (Agilent Technologies, USA) at 65 °C for 17 h. After washing and drying by nitrogen gun blowing, microarrays were scanned with an Agilent microarray scanner (Agilent Technologies, USA) at 535 nm for Cy3. Scanned images were analyzed by Feature extraction10.5.1.1 software (Agilent Technologies, USA), an image analysis and normalization software used to quantify signal and background intensity for each feature.

### Electrophysiological studies of differentiated eGFP+ cardiomyoblasts

FACS-purified Nkx2.5 enh-eGFP+ cells, isolated from 7–10 day-old neonatal hearts were either cultured alone or cocultured with: embryonic day 10 CMs (eCMs, in differentiation medium), smooth muscle cells (SMCs), mouse embryonic fibroblasts (MEFs), or endothelial cells (ECs), for 8 days. For all *in vitro* studies, we used 0.1% gelatin for coating culture substrates. Prior to electrophysiological studies, cocultured eGFP+ cells were dispersed into single cells by collagenase treatment and replated onto 1 cm-diameter round coverslips. Cells on coverslips were bathed in extracellular solution containing 140 mM NaCl, 2.8 mM KCl, 2 mM CaCl_2_, 2 mM MgCl_2_, 10 mM HEPES, and 10 mM glucose, at pH 7.4. Patch electrodes were filled with an intracellular solution containing 140 mM potassium gluconate, 10 mM NaCl, 2 mM MgCl_2_, 10 mM HEPES, 1 mM EGTA, 4 mM Mg-ATP, and 0.3 mM Na-GTP, at pH 7.3, hence, giving resistances of ~2–5 MΏ. Spontaneous CM action potentials were recorded at room temperature in current clamp mode as previously described [20].

### PCR and quantitative PCR analysis of gene expression

To determine the Cre excision status of LoxP flanked stopper sequence in the ROSA26^FS^LacZ allele of Nkx2.5 enh-eGFP+ cells, eGFP+ cells from the digested hearts of α-MHC–Cre, doxycycline-regulated Nkx2.5 enh-Cre, WT1-CreERT2, and Tie2-Cre transgenic mice were purified by FACS and cultured briefly before their cellular genomic DNA was isolated using the Gentra Puregene kit (Qiagen). These purified DNA samples were PCR amplified with ROSA26 locus-specific primers for the presence of Cre-mediated excision (i.e. 1Lox). The primer sequences are TGG CTT ATC CAA CCC CTA GA (forward), and GTT TTC CCA GTC ACG ACG TT (reverse). Amplification of the HPRT locus was used as an internal PCR control.

For quantitative analysis of gene expression, FACS-purified eGFP+ cells from freshly isolated and collagenase digested hearts were lysed with Trizol (Invitrogen) and stored at −80 °C. Total RNA from each sample was purified from cell lysate using the SV Total RNA kit (Promega). cDNA was made using iScript cDNA synthesis kit (BioRad). Quantitative PCR was performed using the Mastercycler EP Realplex system (Eppendorf) with SYBR Green substrate (BioRad) for 40 cycles.

### Data analysis

Numerical data are presented as mean ± SEM. Statistical significance was performed using a two-tailed paired t-test with equal variance. Correlation between groups was assessed with Pearson correlation coefficients (R). Values of *p* < 0.05 were considered statistically significant.

## Electronic supplementary material

Supplementary Information
