# Supplementary material for: Nkx2.5+ Cardiomyoblasts Contribute to Cardiomyogenesis in the Neonatal Heart
Source: Sci Rep. 2017 Oct 3;7:12590. doi: 10.1038/s41598-017-12869-4 (PMC5626718; doi:10.1038/s41598-017-12869-4)

SUPPLEMENTARY INFORMATION

Nkx2.5+ Cardiomyoblasts Contribute to Cardiomyogenesis in the Neonatal Heart

Vahid Serpooshan[1+], Yuan-Hung Liu[2,3,4+], Jan W. Buikema[1,5], Francisco X. Galdos[1], Orlando Chirikian[1,6], Sharon Paige[1,7], Sneha Venkatraman[1,6], Anusha Kumar[1], David R. Rawnsley[2], Xiaojing Huang[2], Daniël A. Pijnappels[2,8], Sean M. Wu[1,9,10*]

[1] Stanford Cardiovascular Institute, Stanford University School of Medicine, Stanford, CA
[2] Cardiovascular Research Center and [3] Division of Cardiology, Department of Medicine, Massachusetts General Hospital, 185 Cambridge Street, Boston, MA 02114, USA
[4] Section of Cardiology, Cardiovascular Center, Far Eastern Memorial Hospital, New Taipei City, Taiwan
[5] Department of Cardiology, University Medical Center Utrecht, Utrecht, The Netherlands
[6] Biology Program, California State University Channel Islands, Camarillo, CA
[7] Department of Pediatrics, Division of Pediatric Cardiology, Stanford University School of Medicine, Stanford, CA
[8] Department of Cardiology, Leiden University Medical Center, Leiden, the Netherlands
[9] Department of Medicine, Division of Cardiovascular Medicine, and [10] Institute of Stem Cell Biology and Regenerative Medicine, Stanford University School of Medicine, Stanford, CA, USA

+ Authors contributed equally.

*Address for correspondence:
Sean M. Wu, M.D. Ph.D.
G1120A Lokey Stem Cell Building
265 Campus Drive
Stanford, CA 94305
Phone: 650-724-4498
Fax: 650-724-4689
E-mail: smwu@stanford.edu

Figure Legends

Supplemental Figure S1 – IHC analysis of cell proliferation in neonatal cardiac cells. Hearts from Nkx2.5-enh-eGFP mouse were isolated and digested at day 6 after birth. Total heart cells were FACS-sorted into eGFP+ and eGFP- cells and plated onto wells of a 96-well plate. **(A)** At days 1 and 5 of in vitro culture, cells were fixed and immunostained for proliferation markers including Ki67 and pH3. **(B)** Quantification of the percentage of Ki67+ and pH3+ cells within the eGFP+ and eGFP- cell populations.

Supplemental Figure S2 – *In vitro* coculture and differentiation of neonatal Nkx2.5 enh-eGFP+/ROSA26-LacZ cells. **(A)** A schematic diagram of neonatal (P6-P7) eGFP+ cell isolation and 8-day coculture with embryonic cardiomyocytes (eCMs), aortic smooth muscle cells (SMCs), or endothelial cells (ECs) (n = 5). **(B)** Immunocytochemical costaining for cardiac troponin T (cTnT, top row), smooth muscle myosin heavy chain (SM-MHC, middle row), CD31/PECAM (lower row), and LacZ (blue, all panels) in single culture (left column) or coculture with mouse embryonic fibroblasts (MEF, middle column), or eCMs (top right panel), SMCs (middle right panel), or ECs (lower right panel). **(C)** Quantification of the percentage of LacZ+ cells that express the indicated lineage marker.

Supplemental Figure S3 – Determination of the cell lineage origin of neonatal Nkx2.5 enh-eGFP+ cells. **(A)** A schematic diagram of mouse breeding and FACS-based purification of eGFP+ cells from single, double, or triple transgenic mice carrying the indicated Cre lineage marker, $ROSA26^{FS}LacZ$ Cre reporter, and Nkx2.5 enh-eGFP transgene (n = 3 for each Cre line). **(B)** Genomic PCR analysis for the excision of LoxP-flanked sequence in the $ROSA26^{FS}LacZ$ allele in double or triple transgenic mice. For WT1-CreET2 epicardial lineage labeling, tamoxifen was administered to time-mated pregnant female mice at embryonic days 9.5, 11.5, and 13.5.

Supplemental Figure S4 – (A) FACS analysis of P0 Nkx2.5 enh-eGFP+ cells costained with cTnT. Hearts from Nkx2.5 enh-eGFP mouse were isolated at day 0 after birth and digested and immediately fixed for immunostaining with antibodies to cTnT and analyzed on flow cytometer. Note that cTnT+ cells represent about 0.01% of total heart cells and are a negligible proportion of all eGFP+ cells. (B) FACS analysis of control (unstained) total heart cells collected from P0 Nkx2.5 enh-eGFP mouse.

Figures

Supplementary Figure S1

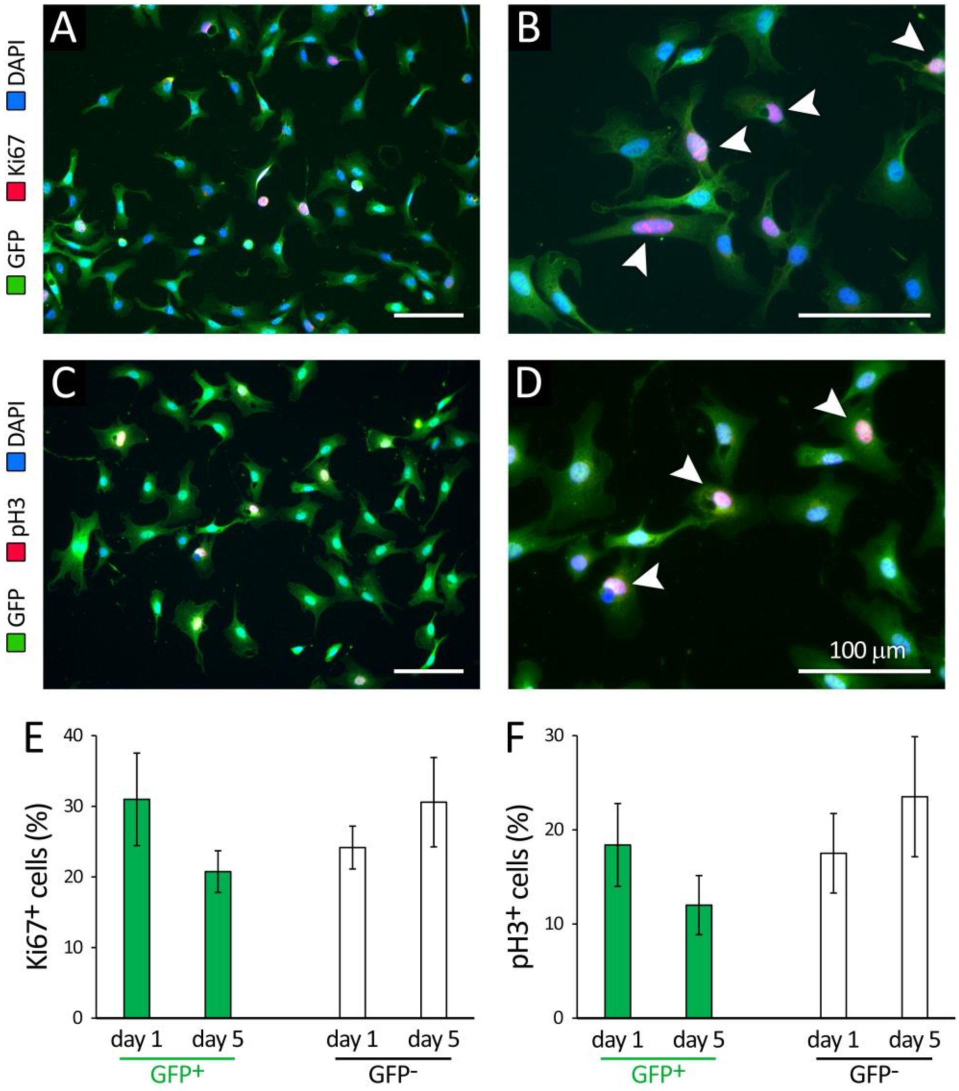

Supplementary Figure S2

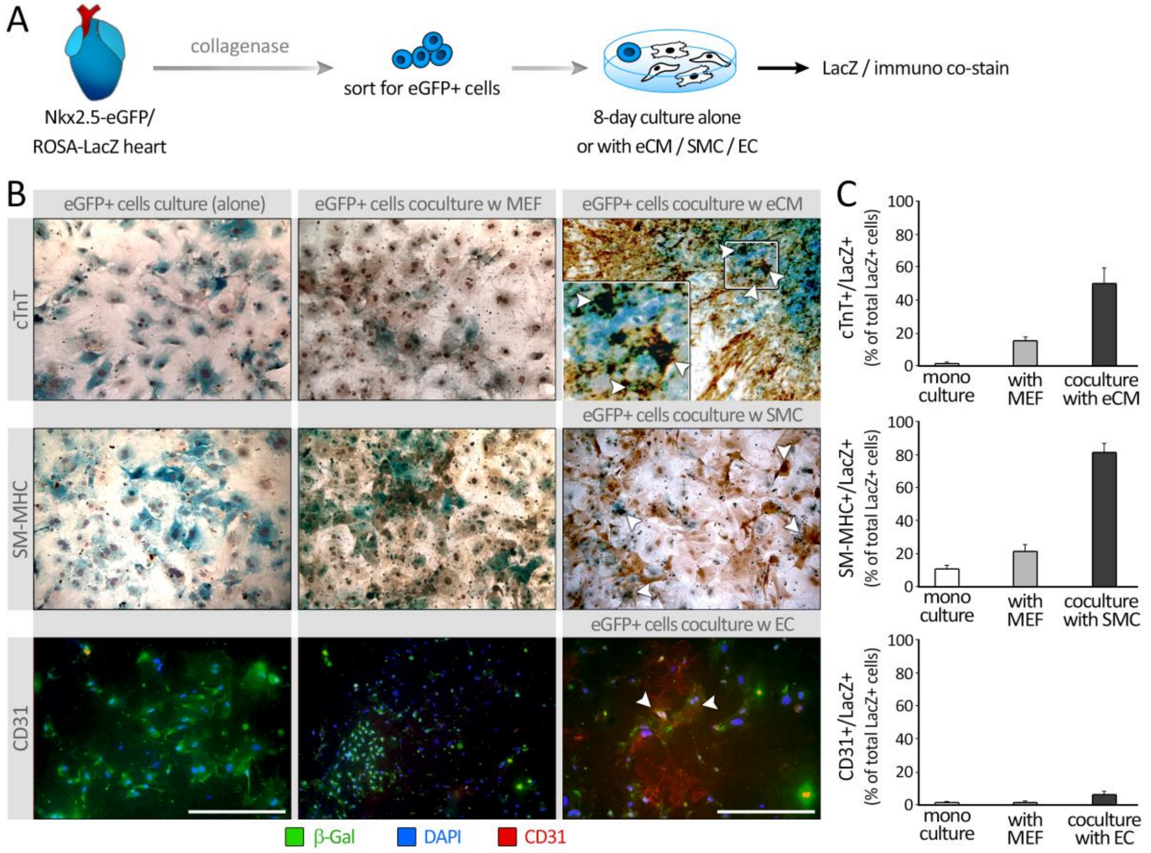

Supplementary Figure S3

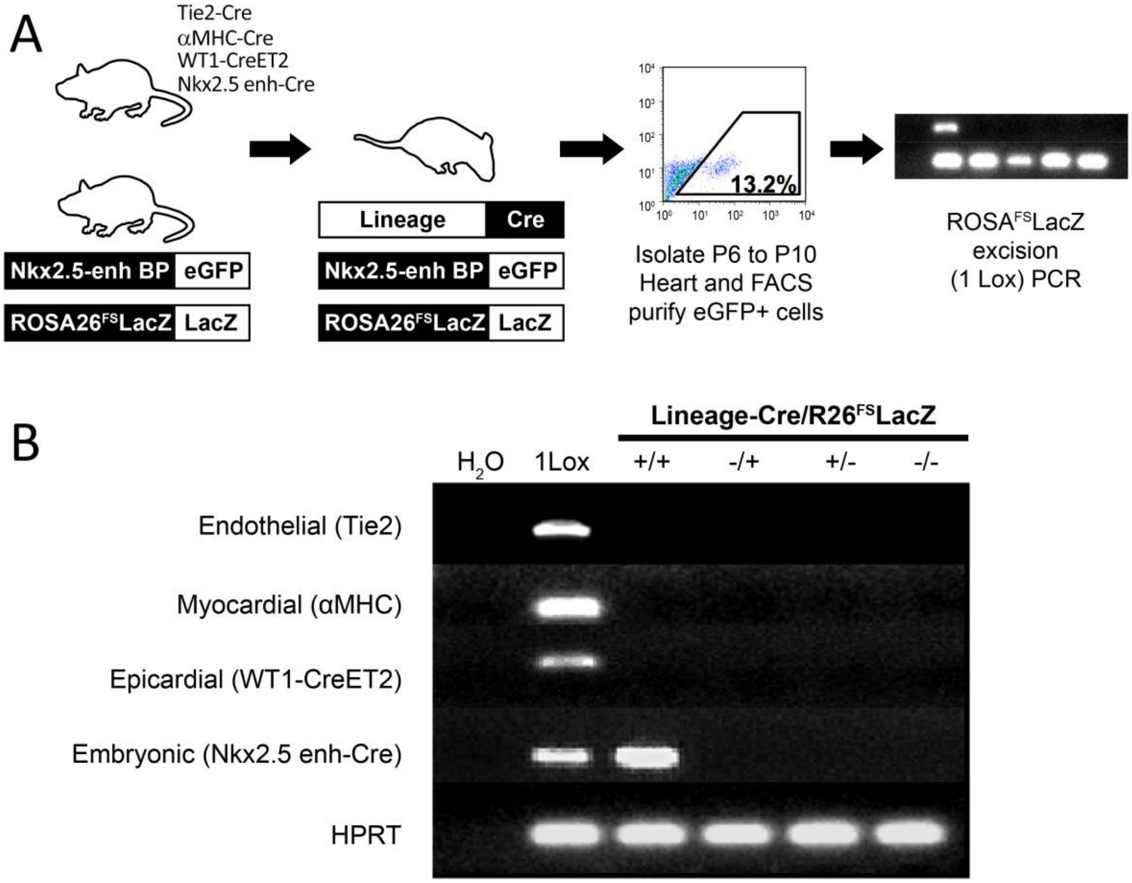

Supplementary Figure S4

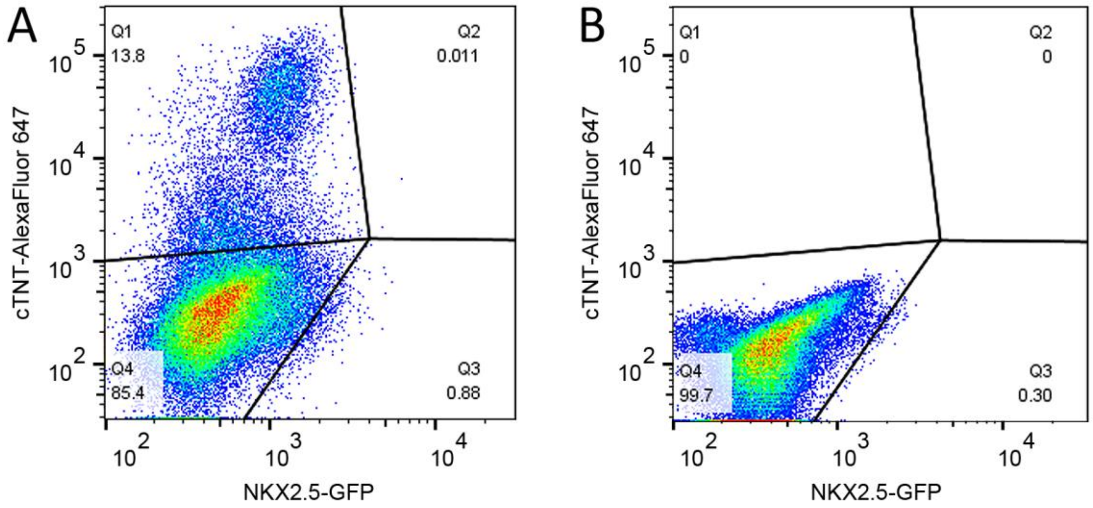

Supplement: Supplementary file 1 — Supplementary Information [file 41598_2017_12869_MOESM1_ESM.pdf]
